# Functional Analysis of Human *GBA1* Missense Mutations in *Drosophila*: Insights into Gaucher Disease Pathogenesis and Phenotypic Consequences

**DOI:** 10.3390/cells13191619

**Published:** 2024-09-27

**Authors:** Aparna Kuppuramalingam, Or Cabasso, Mia Horowitz

**Affiliations:** 1 Shmunis School of Biomedicine and Cancer Research, Faculty of Life Sciences, Tel Aviv University, Tel Aviv 69978, Israel; aparnak@mail.tau.ac.il (A.K.); orcaba@gmail.com (O.C.); 2 Sagol School of Neuroscience, Faculty of Life Sciences, Tel Aviv University, Tel Aviv 69978, Israel

**Keywords:** Gaucher disease, *GBA1*, acid-β-glucocerebrosidase, glucosylceramide, *Drosophila*, ambroxol

## Abstract

The human *GBA1* gene encodes lysosomal acid β-glucocerebrosidase, whose activity is deficient in Gaucher disease (GD). In *Drosophila*, there are two *GBA1* orthologs, *Gba1a* and *Gba1b*, and *Gba1b* is the bona fide GCase encoding gene. Several fly lines with different deletions in the *Gba1b* were studied in the past. However, since most GD-associated *GBA1* mutations are point mutations, we created missense mutations homologous to the two most common GD mutations: the mild N370S mutation (D415S in *Drosophila*) and the severe L444P mutation (L494P in *Drosophila*), using the CRISPR-Cas9 technology. Flies homozygous for the D415S mutation (dubbed D370S hereafter) presented low GCase activity and substrate accumulation, which led to lysosomal defects, activation of the Unfolded Protein Response (UPR), inflammation/neuroinflammation, and neurodegeneration along with earlier death compared to control flies. Surprisingly, the L494P (called L444P hereafter) flies presented higher GCase activity with fewer lysosomal defects and milder disease in comparison to that presented by the D370S homozygous flies. Treatment with ambroxol had a limited effect on all homozygous fly lines tested. Overall, our results underscore the differences between the fly and human GCase enzymes, as evidenced by the distinct phenotypic outcomes of mutations in flies compared to those observed in human GD patients.

## 1. Introduction

Gaucher disease (GD), a lysosomal storage disorder, results from the defective activity of lysosomal acid β-glucocerebrosidase (GCase, EC 3.2.1.4 https://enzyme.expasy.org/EC/3.2.1.45, accessed on 3 April 2023), encoded by the *GBA1* gene. This defective activity primes glucosylceramide (GlcCer) [1,2] and glucosylsphingosine (GlcSph) accumulation [2,3,4,5]. There are three major GD types: type 1 (GD1), which is the most prevalent form and does not involve neurological symptoms; type 2 (GD2), which is an acute neuronopathic disease with fast deterioration and death within the first few years of life; and type 3 (GD3), a neuronopathic disease with longer survival [6]. Among the 1624 mutations in the *GBA1* gene (https://gnomad.broadinstitute.org, accessed on 3 January 2024), the N370S (6728 A>G) (classical nomenclature; new HUGO nomenclature: N409S) is the most prevalent among Ashkenazi Jewish patients [7], while the L444P variant (7319 T>C) (classical nomenclature; new HUGO nomenclature: L483P) is the most prevalent among non-Ashkenazi patients [8]. Homozygosity or compound heterozygosity for the N370S mutation concludes in the development of GD1, while homozygosity for L444P leads to the development of GD3 [7,9].

GCase is a protein of the secretory pathway, which is synthesized on ER-bound polyribosomes, and, following entrance into the ER through the membranal translocons, it undergoes four N-linked glycosylations [10] and folding. Correctly folded GCase traffics to the lysosome through a mannose-6-phosphate (M6P) independent pathway [11]. Mutant GCase variants, recognized as misfolded, are retained in the ER [12]. ER misfolded molecules undergo several refolding attempts by the ER quality control machinery, which, if not successful, undergo retrotranslocation to the cytoplasm, where they are ubiquitinated and degraded in the proteasome in the ER-Associated Degradation (ERAD) process [12,13,14]. ER retention of misfolded molecules creates stress, which leads to activation of the stress response process, known as the Unfolded Protein Response (UPR) [15,16,17].

Removal of misfolded proteins from the ER can be achieved by either chemical or pharmacological chaperones [18]. While chemical chaperones increase the folding capacity of the cell [19], pharmacological chaperones bind to the misfolded molecules and aid their proper folding and exit from the ER [20]. Ambroxol is a known pharmacological chaperone of mutant GCase [21] and has been shown to have an effect in skin fibroblasts that originated from GD patients [22], in mice [23,24], in primates [25], in transgenic GD flies [26], and in humans [27,28,29,30,31,32].

Several GD-like models were established in *Drosophila melanogaster*. *Drosophila* has two *GBA1* orthologs on chromosome 3: *Gba1a* (CG31148) and *Gba1b* (CG31414), of which only *Gba1b* encodes an enzyme with GCase activity [33,34,35,36,37,38,39]. Davis et al. created a deletion of the entire *Gba1b* gene [34]. Flies homozygous for this deletion presented an accumulation of ubiquitinated protein aggregates, neurological dysfunction, and neurodegeneration, which led to a shorter lifespan. In another publication, a *Gba1b* knock-out (KO) and a *Gba1a*-*Gba1b* double KO model were established [35]. Both *Gba1b* KO flies displayed lysosomal deficiencies, progressive age-dependent locomotor deficits, autophagic deficits, synaptic loss, neurodegeneration, and decreased survival. Recently, upregulated inflammation, gut dysfunction, and brain glial activation were documented in the *Gba1b* KO flies [39]. Other groups used an available line containing a Minos insertion in the *Gba1b* gene [36,37]. This truncated allele had no measurable GCase activity, showed a significant GlcCer and GlcSph accumulation, stimulated the UPR, caused inflammation/neuroinflammation, and led to neurodegeneration [37]. Remarkably, in humans, no GCase activity is incompatible with postnatal life [40]; however, the *Gba1b* mutant flies survived to adulthood [37].

In the present study, we generated fly lines harboring the D370S or the L444P point mutations in their *Gba1b* gene and characterized them. The homozygous D370S lines had significantly decreased GCase activity (1–6% of normal GCase), while the L444P homozygous lines presented 20–80% activity of normal GCase. All homozygous lines showed varying degrees of lysosomal abnormalities, activation of UPR, and inflammation/neuroinflammation. The survival of the D370S homozygous lines was significantly shorter than that of lines homozygous for the L444P mutation. Treatment with the pharmacological chaperone ambroxol did not seem to have a significant effect on the mutant flies, most probably due to the limited ability of the drug to bind to the fly GCase.

## 2. Materials and Methods

### 2.1. Antibodies

The following primary antibodies were used in this study: mouse monoclonal anti-myc antibody (Cell Signaling Technology, Beverly, MA, USA) and mouse monoclonal anti-actin antibody (Sigma-Aldrich, Jerusalem, Israel). The secondary antibody used was Horseradish peroxidase-conjugated goat anti-mouse antibodies (Jackson Immuno Research Laboratories, West Grove, PA, USA).

### 2.2. Plasmids Construction

pcDNA4-*Gba1b* WT-mycHis and pUAST-*Gba1b* WT-mycHis plasmids already existed in the lab [37]. Plasmids containing L444P *Gba1b* and D370S *Gba1b* were constructed by in vitro site-directed mutagenesis of pcDNA4-*Gba1b* WT-mycHis using a Quick-Change site-directed mutagenesis kit (Agilent Technologies, Santa Clara, CA, USA), according to the supplier’s instructions, with primers shown in Table 1. The existence of mutations was confirmed by sequencing. The two mutant EcoRI-XhoI fragments were isolated from the plasmids and inserted into the EcoRI and XhoI sites of the pUAST-mycHis plasmid (Invitrogen Life Technologies Co., Carlsbad, CA, USA) to create pUAST-L444P *Gba1b*-mycHis and pUAST-D370S *Gba1b*-mycHis plasmids.

DpnI, EcoRI, and XhoI were purchased from Thermofisher Scientific (Waltham, MA, USA).

### 2.3. Fly Strains

All experiments were performed with isogenized w1118 as a control (Bloomington *Drosophila* Stock Center, Indiana University, Bloomington, Indiana, USA, no. 5905). Strain harboring a Minos transposable element in *Gba1b* was obtained from Bloomington Stock Center (line no. 23602). The balanced lines used in this study were w; Sco/Cyo; *Gba1b^m^*/*Gba1b^m^* (denoted as *Gba1b^m/m^*) and w; Sco/Cyo; *Gba1b^m^*/Sb (denoted as *Gba1b^m/+^*).

The creation of fly lines containing the L444P and the D370S mutations in the *Gba1b* gene was performed by GenetiVision (Houston, TX, USA). gRNAs and donor construct (Table 2) were injected into embryos of a nos-Cas9 line (Bloomington no. 54591) in which the 3rd chromosome originated from an isogenic w1118 (Bloomington no. 5905). To create the donor construct, a 2741 bp fragment of *Gba1b* spanning nucleotides 526-3267 of the gene (https://www.ncbi.nlm.nih.gov, Accession: NT_033777.3 GI: 671162122, accessed 26 September 2024) was cloned into the pUC57 plasmid in the BsaI restriction site and the point mutations were introduced. In addition, two silent mutations (in green) were designed at the PAM region of the gRNA targets (tcactggccatcgatcacgtttc and ATattggtccaaatgtagggtga) to prevent additional CRISPR/Cas9 system from attacking the established mutant allele.

Individual G0 flies were crossed to w;;TM3, Sb, e/TM6B, e, Tb to produce F1 offspring on chromosome 3. Hundred F1 male offspring over 3rd chromosome balancer were randomly selected and crossed with virgin females of genotype w;;TM3, Sb, e/TM6B, e, Tb. From individual offspring, genomic DNA was extracted and sequenced to identify the heterozygous flies that carried the desired mutations. To eliminate off-target mutations, which are not tightly linked to the *Gba1b* gene, heterozygous flies were crossed with w1118 flies for five generations, and sequencing was performed using the non-lethal genotyping as described elsewhere [41]. Briefly, a foreleg was sectioned from an anesthetized fly and was dissolved in 10 µL of squishing buffer [10 mM Tris-HCl, 1 mM EDTA, 25 mM NaCl, 10 µg/mL proteinase K (Sigma Aldrich, Rehovot, Israel)]. Following a 1 h incubation at 37 °C, the region containing the point mutations was amplified using 1 µL of the DNA preparation, 0.5 µM forward and reverse primers (Table 3), and 5 µL of 2X Taq Mix Red HS PCR buffer (PCR Biosystems, Wayne, PA, USA) in total volume of 10 µL. Thermocycler conditions were 95 °C (2 min), and 35 cycles of 95 °C (10 s), 55 °C (20 s), 72 °C (10 s), and another cycle of 72 °C (10 min). The amplified products were then sequenced.

Heterozygous males and females were crossed to obtain homozygous flies.

*Gba1b^m/m^ flies* were described elsewhere [37]. pUAST-L444P *Gba1b*-mycHis and pUAST-D370S *Gba1b*-mycHis (see plasmid construction) were used to establish transgenic lines by BestGene (Chino Hills, CA, USA).

The Da (daughterless)-GAL4 driver line was obtained from Bloomington Stock Center (No. 27608).

All fly strains were grown at 25 °C, unless otherwise stated, and were maintained on a standard cornmeal–molasses medium.

In all experiments, an equal number of males and females was used.

### 2.4. Ambroxol Treatment

Eighty μL of 1 mM ambroxol (Sigma Aldrich, Rehovot, Israel) were poured on top of 12 mL food-containing vials, which were kept at room temperature for at least one day.

### 2.5. Carbobenzoxy-L-Leucyl-L-Leucyl-L-Leucinal (MG-132) Treatment

Eighty μL of 50 μM MG-132 (Calbiochem, San Diego, CA, USA) were poured on top of 12 mL food-containing vials, which were kept at room temperature for at least one day.

### 2.6. Endoglycosidase-H (Endo-H) Assay

Flies (usually 10 flies in each preparation) were homogenized in NP-40 lysis buffer (20 mM Tris-HCl, 100 mM NaCl, 1 mM MgCl_2_, 0.5% NP-40) containing protease inhibitors (10 µg/mL leupeptin, 10 µg/mL aprotinin, and 0.1 mM phenylmethylsulfonylfluoride (Sigma-Aldrich, Rehovot, Israel)). Lysates containing the same amount of protein were subjected to overnight incubation with endo-H (New England Biolabs, Beverly, MA, USA), according to the manufacturer’s instructions, after which a Western blot was performed (for details, see SDS-PAGE and Western blotting).

### 2.7. GCase Activity Assay

Frozen flies (10 flies in each preparation) were lysed in McIlvaine’s buffer (0.1 M citric acid, pH 4.2, 0.2 M Na_2_HPO_4_, 29:21, vol:vol), and protein concentration was determined. Tissue homogenates containing 100 µg of protein were incubated at 37 °C with 8 µM N-[6-[(7-Nitro-2-1,3-benzoxadiazol-4-yl)amino]caproyl]-glucosylceramide (C6-NBD-GlcCer) (Avanti Polar Lipids, Alabaster, AL, USA) in a final volume of 50 µL McIlvaine’s buffer for 1 h. Reactions were terminated by the addition of three volumes of chloroform: methanol (2:1). Lipids were extracted and separated through Thin Layer Chromatography (TLC) as described under “Total lipid extraction” [42]. N-[6-[(7-Nitro-2-1,3-benzoxadiazol-4-yl) amino]caproyl]-Ceramide (C6-NBD-Cer) was identified with an authentic standard (Matreya LLC, State College, PA, USA), using Amersham imager 600 (Amersham, Buckinghamshire, UK).

### 2.8. Total Lipid Extraction

Lipid extraction was essential, as described elsewhere [37]. Briefly, ten flies were lysed in 300 µL of distilled water, and the protein amount was determined. A total of 900 µL chloroform–methanol (2:1) was added. Following mixing and centrifugation, the lower phase was isolated, dried, and dissolved in 20 µL of chloroform–methanol (2:1) isolated [42]. The samples were separated by TLC (Silica gel 60A; Sigma-Aldrich, St. Louis, MO, USA) in chloroform–butanol–ethyl acetate: 0.25% KCl: methanol (25:25:25:9:16, by vol.). The TLC plates were developed with primulin reagent (Sigma-Aldrich, St. Louis, MO, USA) and quantified by ChemiDoc™ XRS (Bio-Rad laboratories, GmbH, Munich, Germany).

### 2.9. Lysotracker Staining and Confocal Imaging

**Suboesophageal ganglion**: Fifteen-day-old adult brains were dissected in PBS and immediately transferred into PBS containing 1 µM of LysoTracker Red DND-99 (Invitrogen, Eugene, OR, USA). The brains were mounted on slides with DAPI-containing mounting medium (GBI labs, Bothell, WA, USA) and imaged within 15 min of dissection [35] with a Leica SP8 Lightning confocal microscope (Leica Microsystems, Wetzlar, Germany) using a 40x objective. For each sample, one control and one mutant brain were imaged side by side with identical settings. Images were quantified using ImageJ software by measuring the pixel intensity of LysoTracker.

**Gut**: Fifteen-day-old adult guts, dissected in PBS, were immediately transferred into PBS containing 1 µM of LysoTracker Red DND-99 and mounted on slides with a DAPI-containing medium (GBI labs, Bothell, WA, USA). The guts were imaged within 10 min of dissection with a Leica SP8 Lightning (Leica Microsystems, Wetzlar, Germany) confocal microscope using a 40x objective. For each sample, one control and one mutant were imaged side by side with identical settings. Images were quantified as above.

### 2.10. SDS-PAGE and Western Blotting

Ten flies were homogenized in NP-40 lysis buffer (20 mM Tris-HCl, 100 mM NaCl, 1 mM MgCl_2_, 0.5% NP-40) containing protease inhibitors (10 µg/mL leupeptin, 10 µg/mL aprotinin, and 0.1 mM phenylmethylsulfonylfluoride (Sigma Aldrich, Rehovot, Israel)). Samples containing the same amount of protein were electrophoresed through 10% SDS-PAGE and electroblotted onto a nitrocellulose membrane (Schleicher and Schuell BioScience, Keene, NH, USA), which interacted with the appropriate antibodies. The blots were developed and incubated with enhanced chemiluminescence detection reagent (Santa Cruz Biotechnology, Dallas, TX, USA) and analyzed using a luminescent image analyzer (ChemiDoc XRS+, Bio-Rad, Hercules, CA, USA).

### 2.11. RNA Preparation

For RNA extraction, adult flies (10 whole flies or 20 heads in each preparation) were frozen in liquid nitrogen and homogenized in TRIzol^®^ Reagent (Life Technologies, Carlsbad, CA, USA). Extraction was performed according to the manufacturer’s instructions.

### 2.12. cDNA Preparation

One microgram of RNA was reverse transcribed with MMLV reverse transcriptase (Promega Corporation, Madison, CA, USA), using an oligo dT primer (Integrated DNA Technologies, Inc., Coralville, IA, USA) in a total volume of 25 µL, at 42 °C for 1 h. Reactions were stopped by incubation at 70 °C for 15 min.

### 2.13. Quantitative Real-Time PCR

Two microliters of cDNA were used for real-time PCR, which was performed using the “power SYBR green QPCR mix reagent” kit (Applied Biosystems, Foster City, CA, USA). The reaction mixture contained 5 µL SYBR green mix, 300 nM of forward primer, and 300 nM of reverse primer in a final volume of 10 µL. Thermal cycling conditions were 95 °C (10 min) and 40 cycles of 95 °C (10 s), 60 °C (20 s), and 72 °C (20 s). The rp49 housekeeping gene was used for normalization, and the relative expression of each gene was calculated by the 2^−ΔΔCT^ method.

All the primers used for qRT-PCR are detailed in Table 4. The forward primer used for amplification of the Xbp1 RNA could anneal only to its spliced form.

### 2.14. Climbing Assay

The climbing behavior of adult flies was measured using a countercurrent apparatus, essentially as described elsewhere [43]. The Climbing Index (CI) was calculated using the following formula: CI (the weighted mean) =  Σ(*mn_m_*)/N, where *m*—number of test vial, *n_m_*—number of flies in the *m*^th^ vial, and N—total number of flies. CI ranged from 1 (min) to 6 (max).

### 2.15. Survival Assay

For each fly strain, 10 vials, each containing 5 males and 5 females, were maintained on food from day one post-eclosion. Fresh food was supplied every other day, and deaths were recorded.

### 2.16. Molecular Dynamics Simulation for Ambroxol Binding

WT *Drosophila Gba1b* was modeled using PDB 2NT0 (PubMed id 17187079) as a template by HHPRED (PubMed id 29258817) and Modeller (PubMed id 24573470). The simulations were conducted with GROMACS version 2018 [44], using the GROMOS43a1 force field. Parameters for ambroxol were calculated using Prodrg (PubMed id 15272157). WT fly GCase with ambroxol was running for 16 ns, and WT without ambroxol was running for 10 ns. RMSD for specific loops around the ambroxol binding site was calculated to show the flexibility of the loops with and without ambroxol during the simulations.

### 2.17. Quantification

For Western blots, the intensity of each band was analyzed using a luminescent image analysis. For immunofluorescence, the fluorescence intensity in each fly brain or each gut (presented by one image) was measured using ImageJ software. Pixel intensity (in arbitrary units) was used to quantify fluorescence in the indicated experiments. All images of a given experiment were exposed and processed identically.

### 2.18. Statistics

Parametric statistical tests were used for all comparisons. One-way or two-way ANOVA analyses were performed, depending on the number of variants, followed by a post hoc Dunnett test, using GraphPad Prism 10.2.3 (GraphPad Software, Inc., San Diego, CA, USA). Differences were considered statistically significant if *p* < 0.05. Kaplan–Meier analysis was performed using the XLSTAT 2022.5.1 software (Addinsoft Inc., New York, NY, USA).

## 3. Results

### 3.1. GCase Activity and Substrate Accumulation in the Gba1b^D370S/D370S^ and Gba1b^L444P/L444P^ Lines

**Figure 1:**
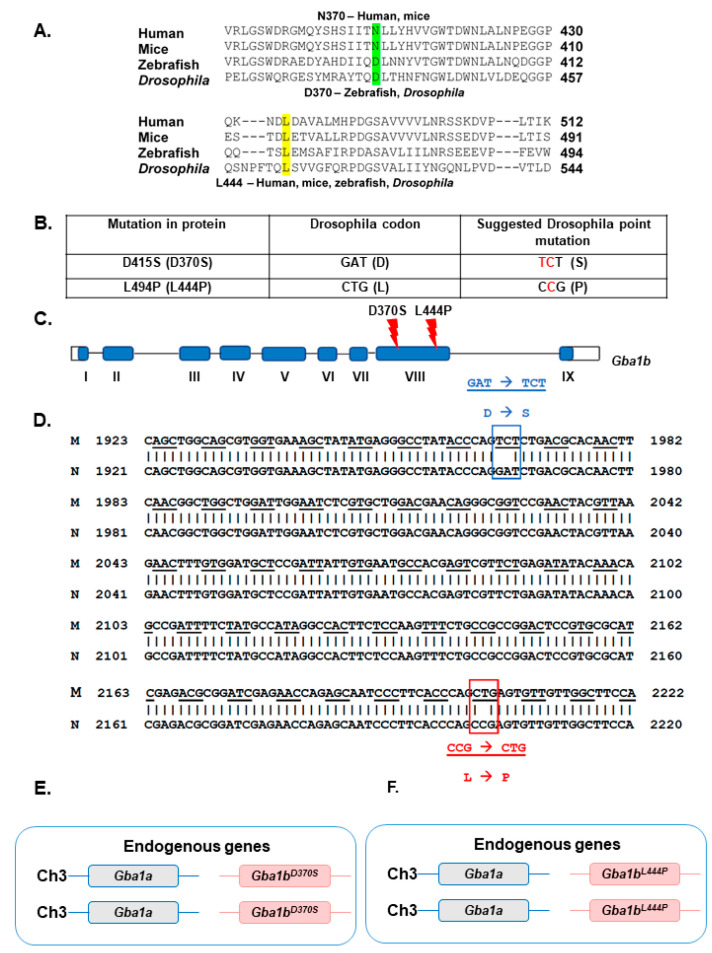
Design of *Drosophila Gba1b^L444P^* and *Gba1b^D370S^* genes. (**A**). Multiple sequence alignment of *GBA1* fragments from different organisms containing the two amino acids that were mutated in the present study. The N370 is highlighted in green, and the L444 is highlighted in yellow. (**B**). The original and the established nucleotide sequence of the mutated amino acids. Highlighted in red are the mutated nucleotides. (**C**). Shown in red is the exon localization of the mutated amino acids. (**D**). Comparison between *Gba1b* fragments containing either the mutant (M) or the normal (N) sequence based on non-lethal genotyping. Boxed in blue are the nucleotide changes introduced to obtain the D370S (D415S) mutation, and in red are the nucleotide changes introduced to obtain the L444P (L494P) mutation. (**E**). Schematic representation of the *Gba1b* region on chromosome 3 of the homozygous *Gba1b^D370S/D370S^* line generated. (**F**). Schematic representation of the *Gba1b* region on chromosome 3 of the homozygous *Gba1b^L444P/L444P^* line generated.

The two most common *GBA1* mutations among GD patients, the N370S and the L444P, were introduced into the fly ortholog, *Gba1b*, using the CRISPR-Cas9 technology, resulting in the generation of several *Gba1b^L444P/+^* and *Gba1b^D370S/+^* lines (Figure 1A–E). The existence of the mutations was confirmed by sequencing the entire endogenous D370S or the L444P-containing genes, and homozygous lines were established by crossings.

**Figure 2:**
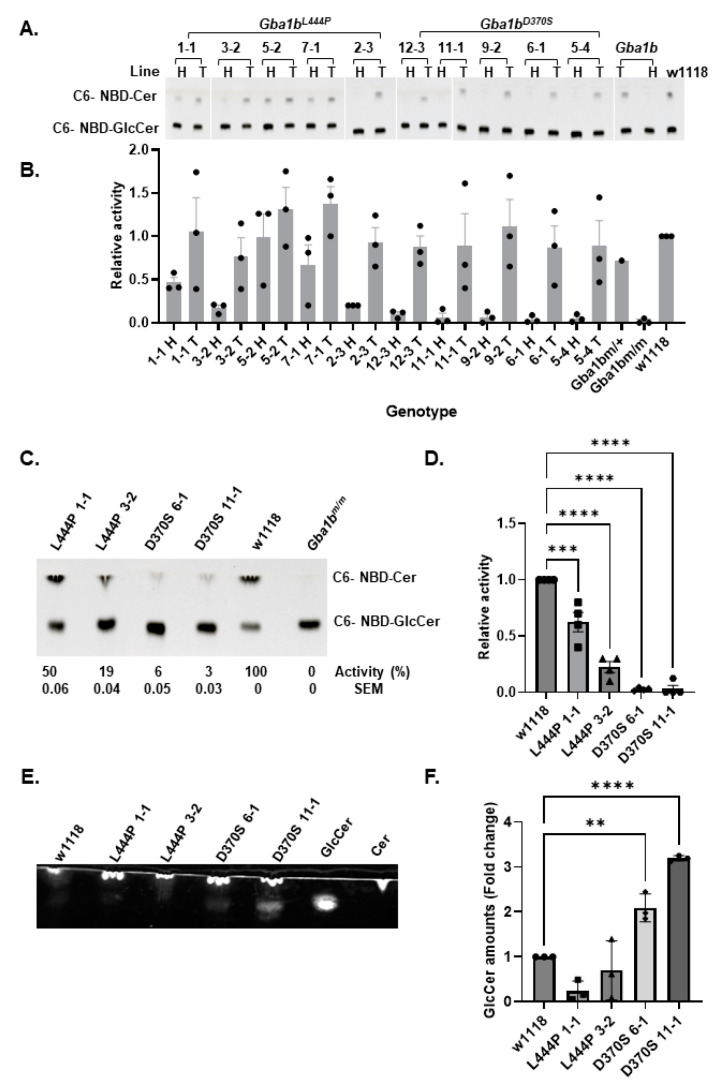
Decreased GCase activity and substrate accumulation in the mutant fly lines. (**A**). GCase activity was measured in 50 µg protein lysates prepared from 2-day-old *Gba1b^L444P/L444P^*, *Gba1b^D370/D370SS^* (homozygous—H) lines, and *Gba1b^D370/+^*, *Gba1b^L444P/+^* (heterozygous—T) flies, as well as from *Gba1b^m/+^* (T), *Gba1b^m/m^* (H), and w1118 lines, as detailed in the Methods section. The activity level of w1118 was considered 1. (**B**). The quantification of (**A**) is shown as the average ± standard error. (**C**). TLC analysis of GCase activity of the four selected homozygous lines (D370S-*Gba1b^D370/D370SS^*; L444P-*Gba1b^L444P/L444P^)*. (**D**). The quantification of (**C**) is shown as the average ± standard error. One-way ANOVA was used to calculate the significance of the results. (**E**). TLC plate showing substrate accumulation in lipid extracts prepared from 22-day-old homozygous flies (D370S-*Gba1b^D370/D370SS^*; L444P-*Gba1b^L444P/L444P^*). (**F**). Quantification of results as shown in (**E**). The results are presented as average ± standard error. One-way ANOVA was used to calculate the significance of the results. ** *p* < 0.01, *** *p* < 0.001, **** *p* < 0.0001. SEM—standard error. Each dot denotes an independent experiment.

GCase activity was tested in lysates of heterozygous and homozygous flies from different lines using the artificial substrate C6-NBD-GlcCer [45,46,47]. It ranged between 20 and 80% of WT activity for the *Gba1b^L444P/L444P^* lines, while it was only 0–6% of WT activity for the *Gba1b^D370S/D370S^* lines (Figure 2A,B). Based on the activity, four homozygous lines were chosen for further experiments: lines 1-1 and 3-2 for *Gba1b^L444P/+^* flies, and lines 6-1 and 11-1 for *Gba1b^D370S/+^* flies, and their activity was re-measured for reassurance (Figure 2C,D). GCase activity was also tested in the selected lines using 4-Methylumbelliferyl-β-D-glucopyranosidase (4-MUG) as a substrate [48]. Activity tested with 4-MUG was consistently higher than that presented using the C6-NBD-GlcCer assay (Supplementary Figure S1A). Moreover, GCase activity was detected in flies homozygous for a 133 C-terminal amino acids deletion of the *Gba1b* gene, which was not expected to have any activity [37]. Based on these results, we abandoned the 4-MUG assay in further experiments.

To determine the amount of substrate accumulated, total lipids extracted from *Gba1b^L444P/L444P^* and *Gba1b^D370S/D370S^* flies were analyzed by TLC. Substrate accumulation was evident only in the *Gba1b^D370S/D370S^* fly lines (Figure 2E,F). A temperature shift to 29 °C induces stress in the flies and makes the pathogenic signs more visible [49,50]. We, therefore, analyzed lipids in flies that were grown at 29 °C. Again, substrate accumulation was detected only in the homozygous *Gba1b^D370S/D370S^* fly lines (Supplementary Figure S1B,C). No GlcCer accumulation was noticed in the *Gba1b^L444P/L444P^* flies, most probably due to the limited sensitivity of the analysis used.

### 3.2. Altered Lysosomal Morphologies in Mutant Flies

**Figure 3:**
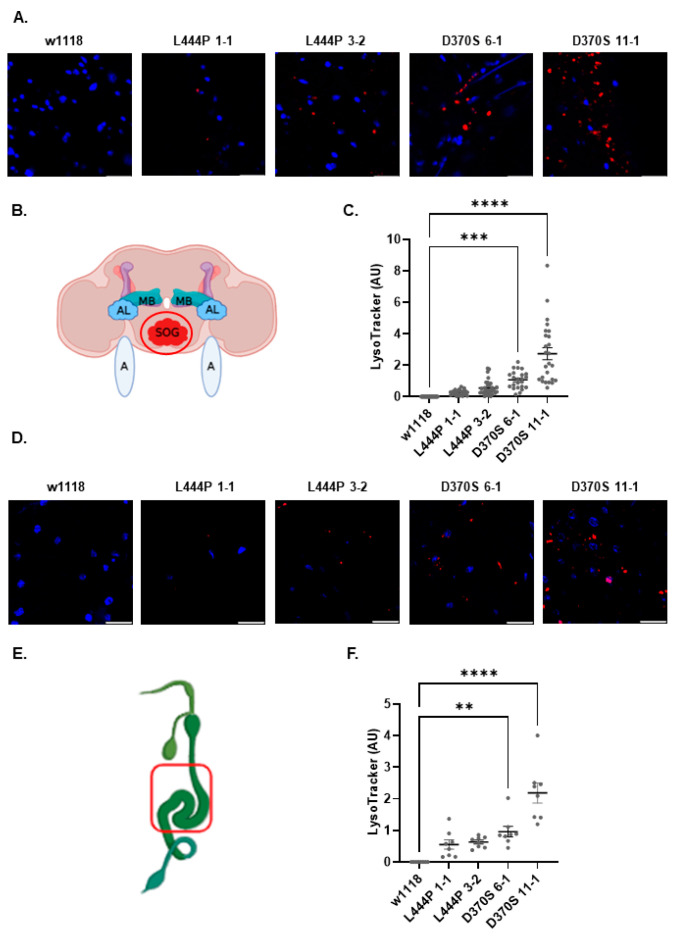
Lysosomal abnormalities in the homozygous mutant flies. (**A**). Confocal images of the suboesophageal ganglion region in the brains of control w1118, *Gba1b^L444P/L444P^* lines 1-1 and 3-2, and *Gba1b^D370S/D370S^* lines 6-1 and 11-1 flies, at 15 days post-eclosion. Red—LysoTracker, Blue—DAPI. (**B**). Graphical presentation of the *Drosophila* brain was created using BioRender. MB—mushroom body, AL—anntenal lobe, SOG—suboesophageal ganglion, A—anntena. The imaged region is circled in red. (**C**). Quantification of LysoTracker intensity in images like the one shown in (**A**). The results are presented as an average ± standard error for 25 different brains for each line. Significance was calculated using one-way ANOVA. (**D**). Confocal images of the gut region of w1118, *Gba1b^L444P/L444P^* lines 1-1 and 3-2, and *Gba1b^D370S/D370S^* lines 6-1 and 11-1 flies at 15 days post-eclosion. Red—LysoTracker, Blue—DAPI. (**E**). Graphical presentation of the *Drosophila* gut. The image was taken from BioRender, and the imaged region is boxed in red. (**F**). Quantification of LysoTracker intensity in images like the one shown in (**D**). The results are presented as an average ± standard error for 7 different guts for each line. Significance was calculated using one-way ANOVA. ** *p* < 0.01, *** *p* < 0.001, **** *p* < 0.0001. Each dot denotes an independent experiment.

Previous studies demonstrated an abnormal enlargement of lysosomes in the suboesophageal ganglion [35,37] and guts [39] of mutant flies with partial or complete deletion of their *Gba1b* gene. To investigate possible lysosomal abnormalities in the *Gba1b^L444P/L444P^* and the *Gba1b^D370S/D370S^* mutant flies, their brains and guts were stained with LysoTracker and visualized. Different amounts, sizes, and intensities of lysosomes were observed in the suboesophageal ganglion (Figure 3A–C) and in the gut (Figure 3D–F) of all tested lines in comparison to that seen in control w1118 flies. There was a direct correlation between the decrease in GCase activity and the amount and size of the detected lysosomes. Thus, in the *Gba1b^L444P/L444P^* flies, the number of stained lysosomes was lower, and their size was smaller in comparison to those perceived in the two *Gba1b^D370S/D370S^* lines.

### 3.3. ER Retention and ERAD of the Mutant Gba1b Variants and Activation of UPR

**Figure 4:**
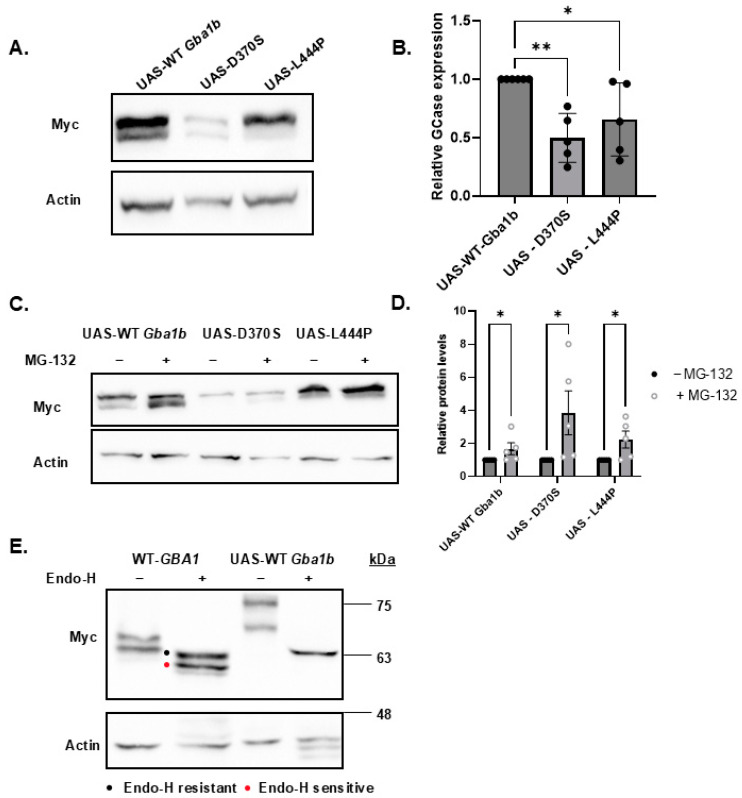
ERAD of the mutant *Gba1b* variants. (**A**). A total of 60 µg of protein lysates, prepared from 2-day-old flies expressing the WT-*Gba1b* (UAS-WT *Gba1b*), D370S (UAS-D370S), and L444P (UAS-L444P) mutants under the Da-GAL4 driver, were electrophoresed through SDS-PAGE and the corresponding blots were interacted with anti-myc antibody to visualize the GCase proteins and with anti-actin antibody, as a loading control. (**B**). Quantification of results as presented in (**A**). The results are presented as average ± standard error. One-way ANOVA was used to determine the statistical significance of the results. (**C**). Protein lysates (60 µg), prepared from 22-day-old mutant flies described in (**A**), were processed as specified in (**A**). (**D**). Quantification of results as presented in (**C**). The results are presented as average ± standard error. Analysis was performed as explained in (**B**). (**E**). Protein lysates (60 µg), prepared as in (**A**) and treated with endo-H were subjected to electrophoresis and blotting as in (**C**). The blots interacted with anti-myc antibody to visualize the GCase proteins and with anti-actin antibody as a loading control. * *p* < 0.05, ** *p* < 0.01. Each dot denotes an independent experiment.

Misfolding and ERAD were documented for the N370S and the L444P human mutant GCase variants [12,26,51]. Since there are no anti-fly GCase-specific antibodies, we could not directly test whether the fly D370S and L444P GCase variants are misfolded and undergo ERAD. To overcome this obstacle, we established transgenic flies, expressing the two mutant fly variants, and tested their steady-state amount. The results indicated that the steady-state level of *Drosophila* D370S and L444P mutant GCase variants was lower than that of the WT-*Gba1b* protein (Figure 4A,B), with the D370S variant having a lower level than the L444P variant. These results indicated that the N370S mutant protein undergoes more ERAD than the L444P mutant variant. To directly test it, the possible stabilization of both mutant proteins by the proteasome inhibitor MG-132 was examined. The results indicated stabilization of both mutant proteins upon MG-132 treatment, strongly indicating ERAD of both of them (Figure 4C,D).

To further understand the processing of the mutant *Gba1b* variants in *Drosophila*, an endoglycosidase-H (endo-H) assay was performed. Endo-H recognizes N-glycan trees with more than four mannose residues [52]. Therefore, it distinguishes between high mannose N-glycan complexes (that are predominantly found in proteins in the ER and are endo-H sensitive) and the complex glycans (that are part of proteins in the *trans*-Golgi/lysosomes and therefore are endo-H resistant) [12,53]. A preliminary endo-H assay was performed on the lysates of flies expressing the normal fly *Gba1b* or the human normal *GBA1*. The results (Figure 4E) documented two human WT GCase peptides, and upon endo-H treatment, the upper band showed a decrease in its molecular weight, indicating a cleavage of the N-glycan trees. Therefore, the upper band was most probably endo-H sensitive and in the ER. On the other hand, the lower peptide was endo-H resistant and, therefore, a lysosomal form. Contrary to these results, the *Drosophila* GCase was completely endo-H sensitive. Remarkably, the majority (69.3%) of N-glycans in *Drosophila* are highly mannosylated [54], which, most probably, explains why the normal fly *Gba1b*-encoded GCase was endo-H sensitive. Based on this result, we did not pursue the endo-H sensitivity assay on the fly-encoded mutant GCase variants.

**Figure 5:**
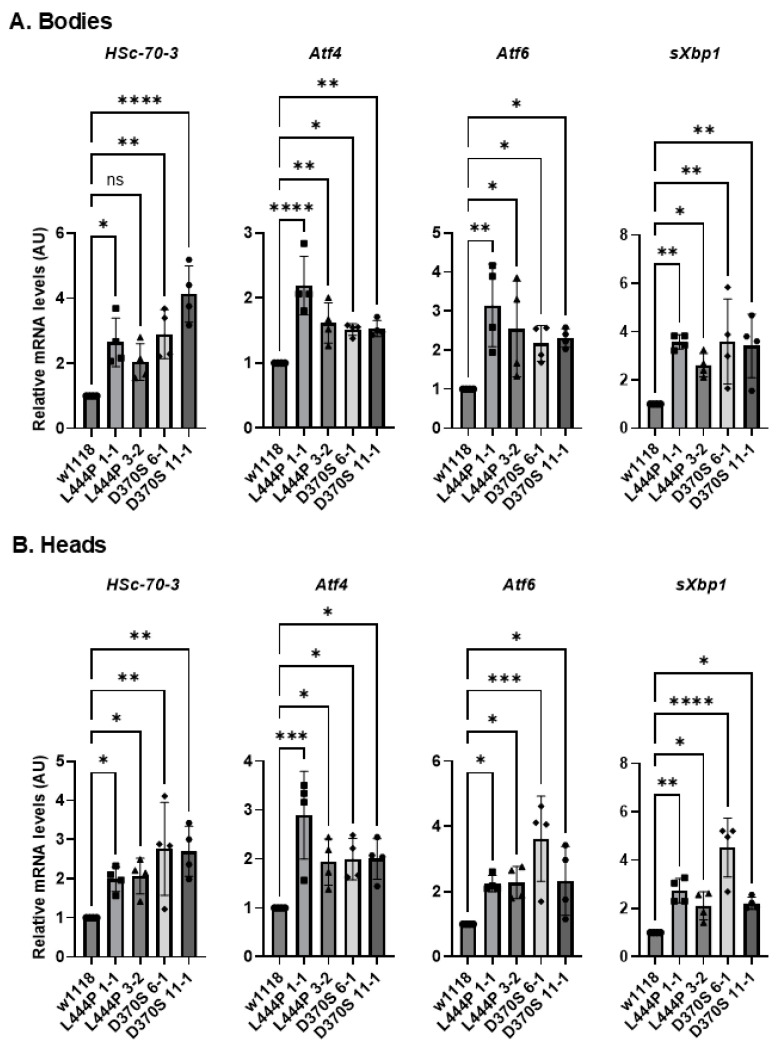
UPR activation in the mutant flies. (**A**). mRNA levels of UPR markers: HSc-70-3, Atf4, Atf6, and sXbp1 were tested in the bodies (**A**) and heads (**B**) of 22-day-old homozygous *Gba1b^L444P/L444P^* fly lines 3-2 and 1-1 and homozygous *Gba1b^D370S/D370S^* fly lines 6-1 and 11-1. The results are presented as average ± standard error. Each dot represents a triplicate of an independent experiment. One-way ANOVA was used to determine the statistical significance of the results. * *p* < 0.05, ** *p* < 0.01, *** *p* < 0.001, **** *p* < 0.0001, ns—non-significant. Each dot denotes an independent experiment.

UPR is activated due to ER stress, initiated by the chronic retention of misfolded proteins in the ER [15,16]. We have shown in the past that UPR is activated in human-derived GD skin fibroblasts [12,51,55], in white blood cells [55], in *Drosophila* lines expressing human mutant variants [26,51] or in *Gba1b* mutant flies [37]. To test UPR in the *Gba1b^L444P/L444P^* and the *Gba1b^D370S/D370S^* flies, changes in UPR markers were followed in their heads and bodies using qRT-PCR. The UPR parameters chosen to test were Heat Shock-70-3 (HSc-70-3), activating transcription factors 4 and 6 (Atf4, Atf6), and spliced X-box binding protein (sXbp1). A significant elevation of all four tested UPR markers in the bodies and heads of all tested lines was noticed (Figure 5A,B), indicating ER stress due to the presence of misfolded GCase.

Taken together, the results documented ERAD of mutant GCase and UPR activation in all tested homozygous lines.

### 3.4. Activation of Inflammation in the Mutant Flies

**Figure 6:**
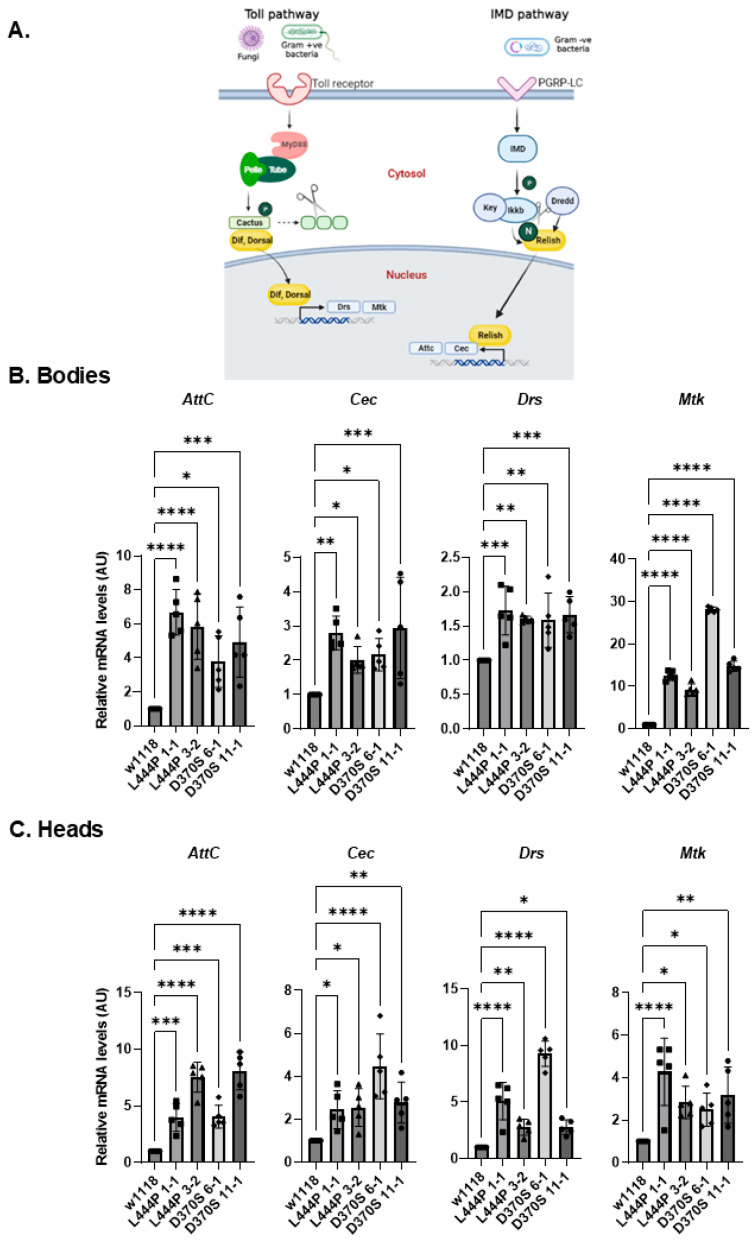
Inflammation and neuroinflammation in the mutant flies. (**A**). The inflammatory pathways in *Drosophila* (created with BioRender). (**B**). mRNA levels of inflammatory markers: AttC, Cec, Drs, and Mtk were tested in the bodies (**B**) and heads (**C**) of 22-day-old *Gba1b^L444P/L444P^* fly lines 3-2 and 1-1 and *Gba1b^D370S/D370S^* lines 6-1 and 11-1. The results are presented as average ± standard error. One-way ANOVA was used to determine the statistical significance of the results. * *p* < 0.05, ** *p* < 0.01, *** *p* < 0.001, **** *p* < 0.0001. Each dot denotes an independent experiment.

Upon ER stress and UPR activation in humans, various inflammatory pathways, such as the NF-kB-IKK and JNK-AP1 pathways, are activated [56]. In *Drosophila*, there are two major pathways associated with immune response activation: the Toll and Imd pathways, which are homologous to the mammalian Toll-like receptor (TLR) and tumor necrosis factor receptor (TNFR) pathways, respectively [57] (Figure 6A). Once activated by Damage-Associated Molecular Patterns (DAMPs) [58], the receptors lead to signaling pathways that result in the translocation of NF-κB homologous proteins: Dorsal in the Toll pathway and Relish in the Imd pathway, from the cytoplasm to the nucleus and initiation of transcription of antimicrobial peptide (AMP) genes in the nucleus. Each pathway is responsible for the transcription of different AMPs.

To test inflammation and neuroinflammation, mRNA levels of four different AMPs, Attacin-C (AttC) and Cecropin (Cec) in the Imd pathway, and Drosomycin (Drs) and Metchnikowin (Mtk) in the Toll pathway, were measured using qRT-PCR analysis.

An increase in all tested inflammatory markers was observed in the heads and bodies of 22-day-old mutant flies (Figure 6B,C), indicating activation of inflammation and neuroinflammation in all of them.

### 3.5. Neurodegeneration and Decreased Lifespan in the Mutant Flies

**Figure 7:**
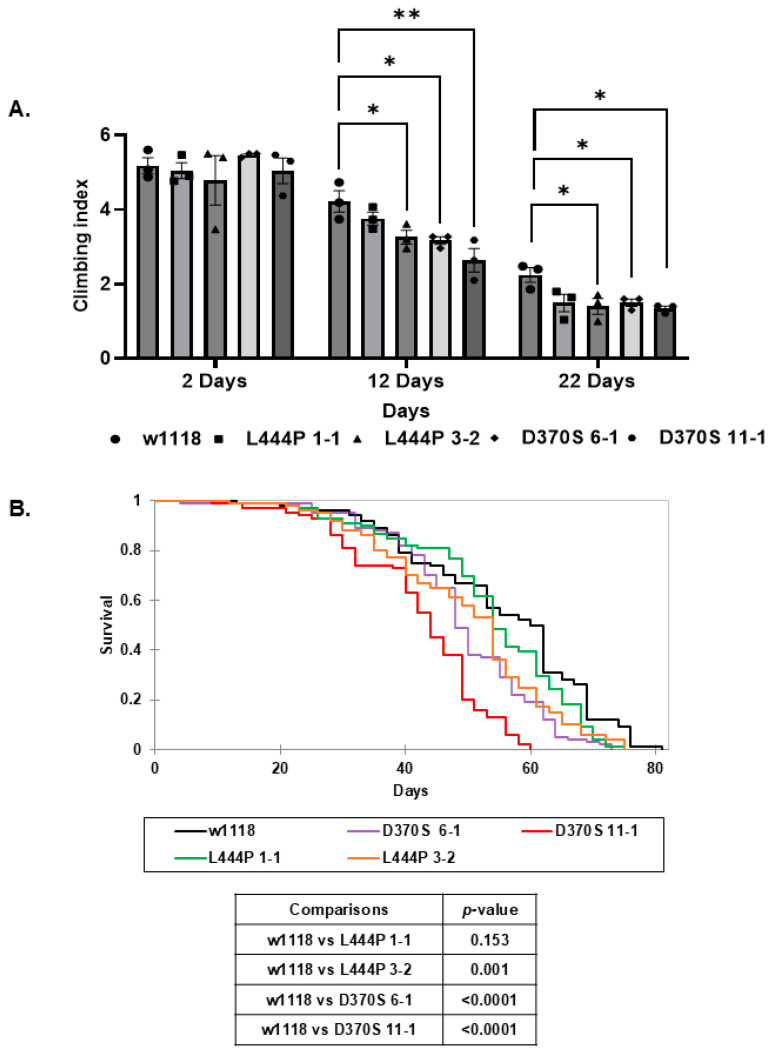
Neuropathology and survival of the mutant flies. (**A**). Thirty flies from *Gba1b^L444P/L444P^* lines 3-2 and 1-1 and *Gba1b^D370S/D370S^* lines 6-1 and 11-1 were tested for their locomotion abilities at 2, 12, and 22 days post-eclosion. Results are presented as average ± standard error. Two-way ANOVA was used to determine the statistical significance of the results. (**B**) Kaplan–Meier curve presenting the survival of 100 control (w1118), homozygous *Gba1b^L444P^* lines 3-2 and 1-1, and *Gba1b^D370S/D370S^* lines 6-1 and 11-1 flies. Below is a table showing the significance measured by Kaplan–Meier’s multiple comparisons. * *p* < 0.05, ** *p* < 0.01. Each dot denotes an independent experiment.

A decline in negative geotaxis (climbing ability) in flies is an indicator of neurodegeneration [59], and therefore, it was tested in the homozygous flies. All the tested fly lines, except for *Gba1b^L444P/L444P^* line 1-1, presented a decrease in their climbing ability, already at day 12, indicating a neurodegeneration detected (Figure 7A). Identical to the negative geotaxis, no significant decline in survival was detected for the *Gba1b^L444P/L444P^* line 1-1 flies, compared with their age-matched w1118 control flies. The other tested lines, *Gba1b^L444P/L444P^* line 3-2 and *Gba1b^D370S/D370S^* lines 6-1 and 11-1, showed a reduced survival compared to that of w1118 flies (Figure 7B).

Taken together, the results show that the low GCase activity in the *Gba1b^D370S/D370S^* flies led to substrate accumulation and lysosomal defects and culminated in neurodegeneration. In the *Gba1b^L444P/L444P^* flies, only line 3-2 showed a neurodegeneration. Regardless of the levels of neurodegeneration, all tested fly lines presented with elevated UPR and inflammation/neuroinflammation.

### 3.6. Partial Rescue of the Mutant-Gba1b Phenotype by Ambroxol

A potential treatment that holds promise for the future of nGD patients is pharmacological chaperone therapy [18]. One such GCase chaperone is ambroxol [21], which was initially shown to increase the amount and lysosomal activity of mutant GCase in GD-derived skin fibroblasts [21,22,60,61] in animal models [24,26,37] and in patients with GD2 [30] or GD3 [27,28].

**Figure 8:**
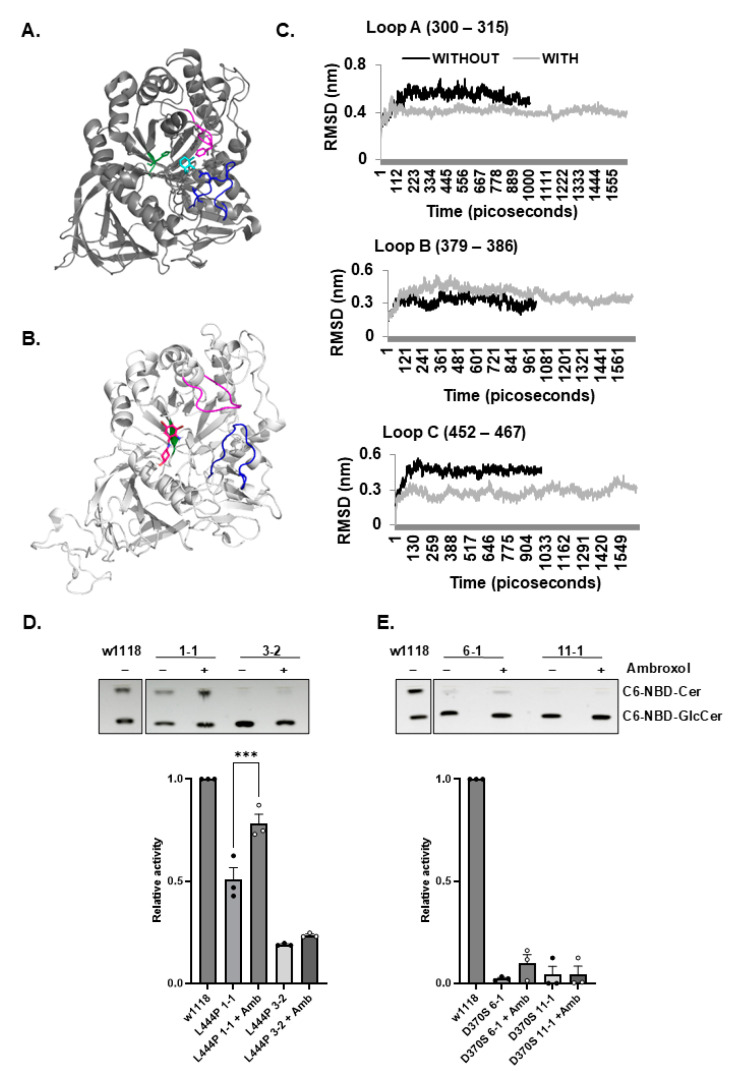
Molecular dynamic simulation of *Gba1b*-encoded GCase with ambroxol. (**A**). X-ray structure of human WT GCase with ambroxol (depicted in cyan) (based on Figure 7B from JBC 2009 [21]). The loops that are stabilized upon formation of ambroxol–GCase complex are pink (loop A), green (loop B), and blue (loop C). (**B**). The predicted *Gba1b* WT model with ambroxol (16 ns stimulation). Ambroxol is painted in dark pink. Loops A, B, and C are colored as in (**B**). (**C**). RMSD stimulation of *Gba1b* GCase with (16 nanoseconds) and without (10 nanoseconds) ambroxol. Each graph shows the RMSD status of a different loop in the *Gba1b*-encoded GCase. Loops A and C are stabilized upon ambroxol binding. (**D**). GCase activity of homozygous *Gba1b^L444P/L444P^* flies (lines 1-1 and 3-2), grown for 22 days with and without ambroxol. GCase activity level of w1118 was considered 1. Results are presented as the average ± standard error. One-way ANOVA was used to calculate the statistical significance. (**E**). GCase activity of the homozygous *Gba1b^D370S/D370S^* flies (lines 6-1 and 11-1), grown for 22 days with and without ambroxol. Activity levels of w1118 with ambroxol were considered 1. Results are represented as the average ± standard error. One-way ANOVA was used to calculate the statistical significance. *** *p* < 0.005. Each dot denotes an independent experiment.

Hydrogen/deuterium exchange mass spectrometry showed that upon binding to ambroxol, amino acid segments 243–249, 310–312, and 386–400 near the active site of human GCase are stabilized. It was predicted that ambroxol interacts with GCase through hydrophobic, π-π interactions and hydrogen bonding [21]. Based on these results, the possible binding of the three corresponding loops in the fly GCase (shown in Table 5) to ambroxol was tested (Figure 8A,B). Root-mean-square deviation of atomic positions (RMSD) stimulation of the three loops of *Gba1b* GCase was tested with and without ambroxol. The results indicated that loops A and C could be stabilized by binding to ambroxol (Figure 8C).

Based on the theoretical binding capability of the fly GCase to ambroxol, we tested the effect of ambroxol on *Gba1b^L444P/L444P^* and the *Gba1b^D370S/D370S^* flies. Since our previous results have not shown any effect of ambroxol on w1118 control flies [37], it was not included in the present study. Upon treatment, there was some (30%) elevation in GCase activity for the *Gba1b^L444P/L444P^* line 1-1 flies and a very slight (less than 5%) elevation for the *Gba1b^L444P/L444P^* line 3-2 flies (Figure 8D) with no measurable change in the *Gba1b^D370S/D370S^* lines 6-1 and 11-1 flies, which could be attributed to the insensitivity of the assay used to detect changes when GCase activity is low (Figure 8E). Based on the activity results, we did not measure changes in the substrate in ambroxol-treated flies.

**Figure 9:**
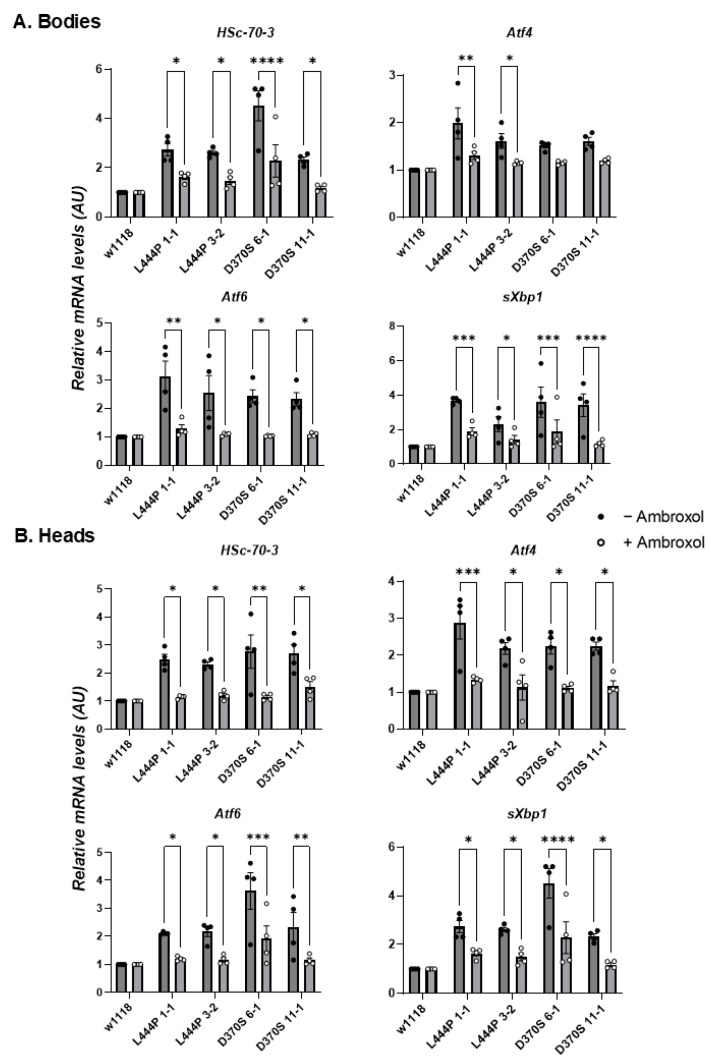
Change in UPR parameters upon ambroxol treatment. (**A**). mRNA levels of UPR markers: HSc-70-3, Atf4, Atf6, and sXbp1 were tested in the bodies (**A**) and heads (**B**) of *Gba1b^L444P/L444P^* lines 3-2 and 1-1 and *Gba1b^D370S/D370S^* lines 6-1 and 11-1 flies that were grown for 22 days with and without ambroxol. The results are presented as average ± standard error. Relative mRNA expression level was calculated using the 2^−ΔΔCT^ method. Two-way ANOVA was used to calculate the statistical significance. * *p* < 0.05, ** *p* < 0.01, *** *p* < 0.001, **** *p* < 0.0001. Each dot denotes an independent experiment.

**Figure 10:**
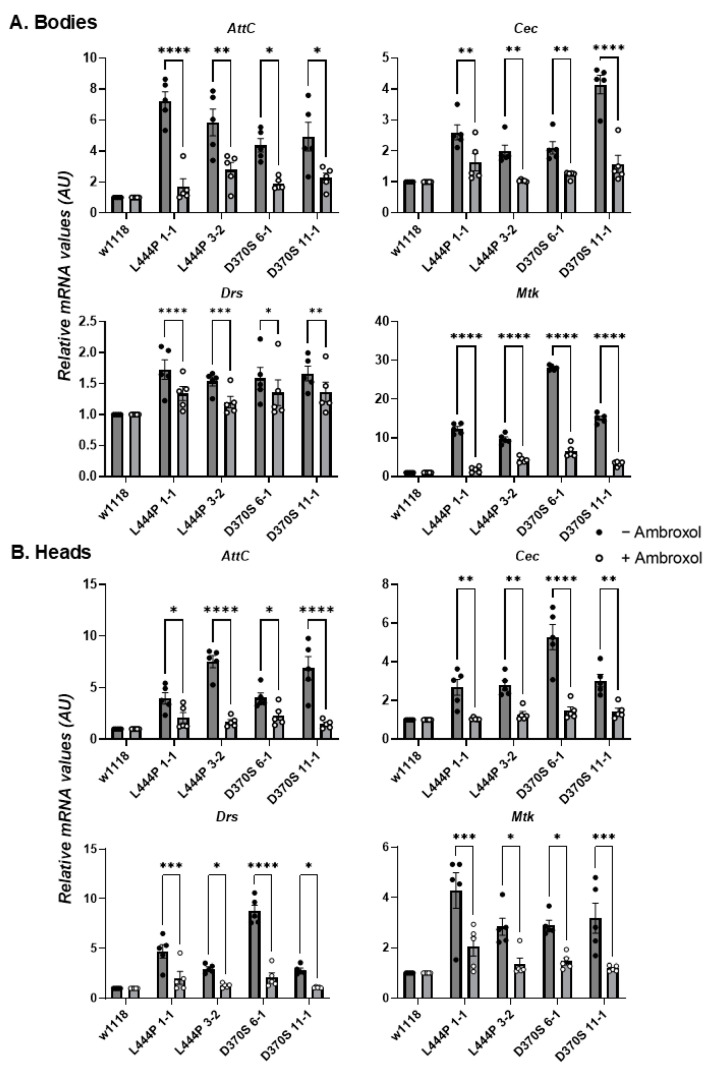
Effect of ambroxol on inflammation/neuroinflammation. (**A**). mRNA levels of inflammatory markers: AttC, Cec, Drs, and Mtk were tested in the bodies (**A**) and heads (**B**) of homozygous *Gba1b^L444P/L444P^* lines 3-2 and 1-1 and *Gba1b^D370S/D370S^* 6-1 and 11-1 flies that were grown for 22 days with and without ambroxol. The results are presented as average ± standard error. Relative mRNA expression level was calculated using the 2^−ΔΔCT^ method. Two-way ANOVA was used to calculate the statistical significance. * *p* < 0.05, ** *p* < 0.01, *** *p* < 0.001, **** *p* < 0.0001. Each dot denotes an independent experiment.

The effect of ambroxol on UPR parameters and inflammation/neuroinflammation was tested as well. A decrease in UPR parameters (Figure 9A,B), as well as in neuroinflammatory/inflammatory parameters (Figure 10A,B), was noted for all mutant flies upon treatment with ambroxol. Concerning the effect of the chaperone on inflammation/neuroinflammation, the results were expected since ambroxol is a known anti-inflammatory compound [62]. Ambroxol did not affect the climbing ability of the mutant flies (Supplementary Figure S2A) nor their lifespan (Supplementary Figure S2B,C), strongly indicating that the flies did not benefit from the decrease in the UPR and in the inflammatory/neuroinflammatory parameters without a significant change in enzyme activity.

To conclude, ambroxol had a minimal effect on both the endogenous *D370S* and the *L444P Gba1b* fly variants.

## 4. Discussion

In the present study, we developed novel knock-in *Drosophila* models for GD by introducing known human GD missense mutations into the fly *Gba1b* gene. *Drosophila* has two *GBA1* orthologs, *Gba1a* and *Gba1b*. *Gba1b* is the fly gene encoding a bona fide GCase enzyme [33,34,35,36,37]. *Gba1a* has no GCase activity [63]. We chose to introduce the two most abundant GD-associated *GBA1* mutations into the fly *Gba1b* gene: the N370S and the L444P mutations. The human leucine 444 is conserved between flies (L494 in the fly, dubbed L444), mice, and zebrafish. The human asparagine 370, which is conserved between humans and mice, appears as aspartic acid in the fly and zebrafish (D415 in the fly, dubbed D370) (Figure 1A). Interestingly, it was recently documented that the human D370 GCase variant is more stable than the N370 variant and has normal activity [64].

From the various flies obtained, we chose two *Gba1b^L444P/L444P^* lines (1-1 and 3-2) and two *Gba1b^D370S/D370S^* lines (6-1 and 11-1) that had lower GCase levels, tested using C6-NBD-GlcCer as a substrate (Figure 2A–D). Remarkably, different *Gba1b^L444P/L444P^* lines with the same point mutation, established by the CRISPR/Cas9 technology using the same parental w1118 line, had significantly different GCase activities. We assume that, as with all CRISPR/Cas9-generated animals, these differences may have resulted from either occasional off-target mutations [65] or genomic variations among the edited animals. However, since the genome sequence of the flies prior to editing is unknown, whole-genome sequencing cannot determine the origin of these variations. Notably, we attempted to mitigate off-target effects by performing five additional crosses of the heterozygous mutant flies with w1118 flies.

When GCase activity was tested using 4-MUG as a substrate, higher activity levels were detected compared to those obtained when using C6-NBD-GlcCer as a substrate (Supplementary Figure S1A). Previous publications describing the knock-out of the fly *Gba1b* gene [34,35] used 4-MUG as a substrate. One study observed a 5–30% activity reduction in heads with no significant reduction in the bodies [34], while the other publication did not indicate GCase levels in the bodies of the mutant flies [35]. The activity levels are surprising since no GCase activity is expected in KO models. Albeit these results, pathological changes were observed in the bodies of the tested KO animals [34,35,39]. Interestingly, Futerman et al. did not use 4-MUG as a substrate to test activity in patient fibroblasts nor in mouse-derived cell lysates but rather the C6-NBD-GlcCer as a substrate [45,47].

All tested mutant lines depicted lysosomal abnormalities in their brain suboesophageal ganglion and in their guts, with more significant abnormalities in the D370S *Gba1b* mutant lines than in the L444P *Gba1b* mutant lines.

Since there are no antibodies available to test the endogenous *Gba1b* protein, D370S *Gba1b-* and L444P *Gba1b*-expressing flies were used to test the ER retention and ERAD of the mutant proteins. While we could not test their ER retention since most fly proteins have highly mannosylated N-glycan trees, which are cleaved by endo-H [54], we could document ERAD of the mutant fly proteins by showing an increase in their protein level upon treatment with MG-132 (Figure 4C,D).

The tested lines presented with increased UPR parameters, inflammation/neuroinflammation, and slight neurodegeneration that affected their survival. However, the *Gba1b^D370S/D370S^* flies presented more severe GD-like symptoms in comparison to the *Gba1b^L444P/L444P^* flies, for which we do not have an explanation. Interestingly, mice homozygous for the N370S mutation also presented a severe disease in comparison to the homozygous L444P mice. While the N370S homozygous mice did not survive beyond the neonatal stage due to epidermal permeability barrier [66], the L444P homozygous mice had about 20% normal GCase activity with no detectable storage of glucosylceramide in the brain and liver. By eliminating the compromised epidermal permeability barrier caused by defective glucosylceramide metabolism in their epidermis [67], L444P homozygous animals were generated with long-term survival. These animals exhibited systemic inflammation without significant GlcCer accumulation in tissues or the presence of Gaucher cells. These results indicated that GCase deficiency, even in the absence of large amounts of sphingolipid storage, can trigger an inflammatory reaction [68].

We tested the effect of ambroxol on mutant fly GCase variants. Our results pointed to a limited effect of ambroxol on the tested fly *Gba1b*-encoded variants, most probably reflecting the limited ability of the drug to bind to the *Drosophila* GCase. A similar partial effect was also observed in a 130 C-terminal amino acid deletion *Gba1b* mutant upon treatment with ambroxol [37]. Though there was an effect of ambroxol on reducing the UPR and inflammatory/neuroinflammatory parameters, it did not lead to improved survival or neurodegeneration. This could indicate that despite the successful transport of the enzyme from the ER to the lysosomes via ambroxol binding and the reduction of ERAD and UPR, its activity in the lysosomes did not significantly increase, resulting in no measurable effect on neurodegeneration or survival.

In summary, consistent with their low enzymatic activity, *Gba1b* mutant flies with the D370S homozygous mutation exhibited activation of UPR, inflammation/neuroinflammation, and lysosomal defects, which led to neurodegeneration evidenced by motor deterioration and reduced survival. In contrast, *Gba1b* mutant flies with the L444P mutation (line 3-2) displayed only minor lysosomal defects and neurodegeneration, while line 1-1 had no detectable neurodegeneration.

The severity of GD signs in the flies was different from the known phenotypes of GD patients homozygous for these mutations. These results strongly suggest that though the human and the fly GCases have the same enzymatic function, the missense mutations tested in the present study confer different changes in the fly compared to those observed in humans.

## Figures and Tables

**Table 1:** The primers used for mutagenesis are shown.

Name	Primers for Plasmid Construction
*L444P Gba1b*	F: 5′–CCCTTCACCCAGCCGAGTGTTGTTGGCTTCCAGCGACC–3′ R: 5′–GGTCGCTGGAAGCCAACAACATCGGCTGGGTGAAGGG-3′
*D370S Gba1b*	F: 5′–GGCCTATACCCAGTCTCTGACGCACAACTTCAACGG–3′ R: 5′–CCGTTGAAGTTGTGCGTCAGAGACTGGGTATAGGCC-3′

**Table 2:** The donor construct and the two sgRNAs used to create mutations in the *Gba1b* gene.

**Donor Construct**	A 2741 bp fragment of the fly *Gba1b* gene in pUC57
**sgRNA 1**	gaaacgtgatcgatggccagtgg
**sgRNA 2**	atattggtccaaatgtagggtgg

**Table 3:** Primers used for amplifying the mutation containing *Gba1b* gene.

Name	Primers for Plasmid Construction
*Gba1b*	F: 5′–GCTATCTGGCTGAACGACAATCTG–3′ R: 5′–CCATTGTAGATTATCAACGCCACAC-3′

**Table 4:** Primers used for qRT-PCR in the present study are depicted.

Name	Primers for qRT-PCR
***rp49***	F: 5′-TAAGAAGCGCACAAAGCACT-3′ R: 5′-GGGCATCAGATATTGTCCCT-3′
***HSc-70-3***	F: 5′-GCTGGTGTTATTGCCGGTCTGC-3′ R: 5′-GATGCCTCGGGATGGTTCCTTGC-3′
***Atf4***	F: 5′-AGACGCTGCTTCGCTTCCTTC-3′ R: 5′-GCCCGTAAGTGCGAGTACGCT-3′
***Atf6***	F: 5′-CGTAATTCCACGGAAGCCCAAC-3′ R: 5′-CGACGGTAGCTTGATTTCTAGAGCC-3′
***sXbp1***	F: 5′-CCGAACTGAAGCAGCAACAGC-3′ R: 5′-GTATACCCTGCGGCAGATCC-3′
***Attc***	F: 5′-CTGCACTGGACTACTCCCACATCA-3′ R: 5′-CGATCCTGCGACTGCCAAAGATTG-3′
***Cec***	F: 5′-CATTGGACAATCGGAAGCTGGGTG-3′ R: 5′-TAATCATCGTGGTCAACCTCGGGC-3′
***Drs***	F: 5′-AGTACTTGTTCGCCCTCTTCGCTG-3′ R: 5′-CCTTGTATCTTCCGGACAGGCAGT-3′
***Mtk***	F: 5′-CATCAATCAATTCCCGCCACCGAG-3′ R: 5′-AAATGGGTCCCTGGTGACGATGAG-3′

**Table 5:** A table showing the amino acid positions of the three loops that are stabilized by ambroxol in human GCase and the parallel positions in the *Drosophila Gba1b*-encoded enzyme.

Loops	Human *GBA1*	*Drosophila Gba1b*
Loop A	243–249	300–315
Loop B	310–312	379–386
Loop C	386–400	452–467

## Data Availability

The original contributions presented in the study are included in the article/Supplementary Materials, further inquiries can be directed to the corresponding author.

## Supplementary Materials

The following supporting information can be downloaded at https://www.mdpi.com/article/10.3390/cells13191619/s1, Figure S1: 4-MUG activity assay and substrate accumulation; Figure S2: Fly pathology upon ambroxol treatment.

## References

[1] Brady R.O., Kanfer J.N., Shapiro D. (1965). Metabolism of glucocerebrosides II. Evidence of an enzymatic deficiency in Gaucher’s disease. Biochem. Biophys. Res. Commun..

[2] Nilsson O., Svennerholm L. (1982). Accumulation of Glucosylceramide and Glucosylsphingosine (Psychosine) in Cerebrum and Cerebellum in Infantile and Juvenile Gaucher Disease. J. Neurochem..

[3] Raghavan S.S., Mumford R.A., Kanfer J.N. (1973). Deficiency of glucosylsphingosine: -Glucosidase in Gaucher disease. Biochem. Biophys. Res. Commun..

[4] Orvisky E., Sidransky E., McKinney C.E., LaMarca M.E., Samimi R., Krasnewich D., Martin B.M., Ginns E.I. (2000). Glucosylsphingosine Accumulation in Mice and Patients with Type 2 Gaucher Disease Begins Early in Gestation. Pediatr. Res..

[5] Dekker N., van Dussen L., Hollak C.E.M., Overkleeft H., Scheij S., Ghauharali K., van Breemen M.J., Ferraz M.J., Groener J.E.M., Maas M. (2011). Elevated plasma glucosylsphingosine in Gaucher disease: Relation to phenotype, storage cell markers, and therapeutic response. Blood.

[6] Brady R.O., Barton N.W., Grabowski G.A. (1993). The Role of Neurogenetics in Gaucher Disease. Arch. Neurol..

[7] Tsuji S., Martin B.M., Barranger J.A., Stubblefield B.K., LaMarca M.E., Ginns E.I. (1988). Genetic heterogeneity in type 1 Gaucher disease: Multiple genotypes in Ashkenazic and non-Ashkenazic individuals. Proc. Natl. Acad. Sci. USA.

[8] Tsuji S., Choudary P.V., Martin B.M., Stubblefield B.K., Mayor J.A., Barranger J.A., Ginns E.I. (1987). A Mutation in the Human Glucocerebrosidase Gene in Neuronopathic Gaucher’s Disease. N. Engl. J. Med..

[9] Wong K., Sidransky E., Verma A., Mixon T., Sandberg G.D., Wakefield L.K., Morrison A., Lwin A., Colegial C., Allman J.M. (2004). Neuropathology provides clues to the pathophysiology of Gaucher disease. Mol. Genet. Metab..

[10] Erickson A.H., Ginns E.I., Barranger J.A. (1985). Biosynthesis of the lysosomal enzyme glucocerebrosidase. J. Biol. Chem..

[11] Rijnboutt S., Aerts H.M., Geuze H.J., Tager J.M., Strous G.J. (1991). Mannose 6-phosphate-independent membrane association of cathepsin D, glucocerebrosidase, and sphingolipid-activating protein in HepG2 cells. J. Biol. Chem..

[12] Ron I., Horowitz M. (2005). ER retention and degradation as the molecular basis underlying Gaucher disease heterogeneity. Hum. Mol. Genet..

[13] Hoseki J., Ushioda R., Nagata K. (2009). Mechanism and components of endoplasmic reticulum-associated degradation. J. Biochem..

[14] Poothong J., Jang I., Kaufman R.J. (2021). Defects in Protein Folding and/or Quality Control Cause Protein Aggregation in the Endoplasmic Reticulum. Prog. Mol. Subcell Biol..

[15] Schröder M. (2006). The Unfolded Protein Response. Mol. Biotechnol..

[16] Kaufman R.J., Back S.H., Song B., Han J., Hassler J. (2010). The unfolded protein response is required to maintain the integrity of the endoplasmic reticulum, prevent oxidative stress and preserve differentiation in *β*-cells. Diabetes Obes. Metab..

[17] Walter P., Ron D. (2011). The Unfolded Protein Response: From Stress Pathway to Homeostatic Regulation. Science.

[18] de la Mata M., Cotán D., Oropesa-Ávila M., Garrido-Maraver J., Cordero M.D., Paz M.V., Pavón A.D., Alcocer-Gómez E., de Lavera I., Ybot-González P. (2015). Pharmacological Chaperones and Coenzyme Q10 Treatment Improves Mutant β-Glucocerebrosidase Activity and Mitochondrial Function in Neuronopathic Forms of Gaucher Disease. Sci. Rep..

[19] Sawkar A.R., Cheng W.-C., Beutler E., Wong C.-H., Balch W.E., Kelly J.W. (2002). Chemical chaperones increase the cellular activity of N370S β-glucosidase: A therapeutic strategy for Gaucher disease. Proc. Natl. Acad. Sci. USA.

[20] Yu Z., Sawkar A.R., Kelly J.W. (2007). Pharmacologic chaperoning as a strategy to treat Gaucher disease. FEBS J..

[21] Maegawa G.H.B., Tropak M.B., Buttner J.D., Rigat B.A., Fuller M., Pandit D., Tang L., Kornhaber G.J., Hamuro Y., Clarke J.T.R. (2009). Identification and Characterization of Ambroxol as an Enzyme Enhancement Agent for Gaucher Disease. J. Biol. Chem..

[22] Bendikov-Bar I., Maor G., Filocamo M., Horowitz M. (2012). Ambroxol as a pharmacological chaperone for mutant glucocerebrosidase. Blood Cells Mol. Dis..

[23] Migdalska-Richards A., Daly L., Bezard E., Schapira A.H.V. (2016). Ambroxol effects in glucocerebrosidase and α-synuclein transgenic mice. Ann. Neurol..

[24] Luan Z., Li L., Higaki K., Nanba E., Suzuki Y., Ohno K. (2012). The chaperone activity and toxicity of ambroxol on Gaucher cells and normal mice. Brain Dev..

[25] Migdalska-Richards A., Ko W.K.D., Li Q., Bezard E., Schapira A.H.V. (2017). Oral ambroxol increases brain glucocerebrosidase activity in a nonhuman primate. Synapse.

[26] Maor G., Cabasso O., Krivoruk O., Rodriguez J., Steller H., Segal D., Horowitz M. (2016). The contribution of mutant *GBA* to the development of Parkinson disease in *Drosophila*. Hum. Mol. Genet..

[27] Narita A., Shirai K., Itamura S., Matsuda A., Ishihara A., Matsushita K., Fukuda C., Kubota N., Takayama R., Shigematsu H. (2016). Ambroxol chaperone therapy for neuronopathic Gaucher disease: A pilot study. Ann. Clin. Transl. Neurol..

[28] Ramadža D.P., Zekušić M., Žigman T., Škaričić A., Bogdanić A., Mustać G., Bošnjak-Nađ K., Ozretić D., Ohno K., Fumić K. (2021). Early initiation of ambroxol treatment diminishes neurological manifestations of type 3 Gaucher disease: A long-term outcome of two siblings. Eur. J. Paediatr. Neurol..

[29] Zhang P., Zheng M.-F., Cui S.-Y., Zhang W., Gao R.-P. (2022). Ambroxol Chaperone Therapy for Gaucher Disease Type I-Associated Liver Cirrhosis and Portal Hypertension: A Case Report. Endocr. Metab. Immune Disord.—Drug Targets.

[30] Aries C., Lohmöller B., Tiede S., Täuber K., Hartmann G., Rudolph C., Muschol N. (2022). Promising Effect of High Dose Ambroxol Treatment on Neurocognition and Motor Development in a Patient With Neuropathic Gaucher Disease 2. Front. Neurol..

[31] Zhan X., Zhang H., Maegawa G.H.B., Wang Y., Gao X., Wang D., Li J. (2023). Use of Ambroxol as Therapy for Gaucher Disease. JAMA Netw. Open.

[32] Istaiti M., Frydman D., Dinur T., Szer J., Revel-Vilk S., Zimran A. (2023). High-Dose Ambroxol Therapy in Type 1 Gaucher Disease Focusing on Patients with Poor Response to Enzyme Replacement Therapy or Substrate Reduction Therapy. Int. J. Mol. Sci..

[33] Suzuki M., Fujikake N., Takeuchi T., Kohyama-Koganeya A., Nakajima K., Hirabayashi Y., Wada K., Nagai Y. (2015). Glucocerebrosidase deficiency accelerates the accumulation of proteinase K-resistant α-synuclein and aggravates neurodegeneration in a *Drosophila* model of Parkinson’s disease. Hum. Mol. Genet..

[34] Davis M.Y., Trinh K., Thomas R.E., Yu S., Germanos A.A., Whitley B.N., Sardi S.P., Montine T.J., Pallanck L.J. (2016). Glucocerebrosidase Deficiency in Drosophila Results in α-Synuclein-Independent Protein Aggregation and Neurodegeneration. PLoS Genet..

[35] Kinghorn K.J., Grönke S., Castillo-Quan J.I., Woodling N.S., Li L., Sirka E., Gegg M., Mills K., Hardy J., Bjedov I. (2016). A *Drosophila* Model of Neuronopathic Gaucher Disease Demonstrates Lysosomal-Autophagic Defects and Altered mTOR Signalling and Is Functionally Rescued by Rapamycin. J. Neurosci..

[36] Kawasaki H., Suzuki T., Ito K., Takahara T., Goto-Inoue N., Setou M., Sakata K., Ishida N. (2017). Minos-insertion mutant of the Drosophila GBA gene homologue showed abnormal phenotypes of climbing ability, sleep and life span with accumulation of hydroxy-glucocerebroside. Gene.

[37] Cabasso O., Paul S., Dorot O., Maor G., Krivoruk O., Pasmanik-Chor M., Mirzaian M., Ferraz M., Aerts J., Horowitz M. (2019). *Drosophila melanogaster* Mutated in its *GBA1b* Ortholog Recapitulates Neuronopathic Gaucher Disease. J. Clin. Med..

[38] Jewett K.A., Thomas R.E., Phan C.Q., Lin B., Milstein G., Yu S., Bettcher L.F., Neto F.C., Djukovic D., Raftery D. (2021). Glucocerebrosidase reduces the spread of protein aggregation in a Drosophila melanogaster model of neurodegeneration by regulating proteins trafficked by extracellular vesicles. PLoS Genet..

[39] Atilano M.L., Hull A., Romila C.-A., Adams M.L., Wildfire J., Ureña E., Dyson M., Ivan-Castillo-Quan J., Partridge L., Kinghorn K.J. (2023). Autophagic dysfunction and gut microbiota dysbiosis cause chronic immune activation in a *Drosophila* model of Gaucher disease. PLoS Genet..

[40] Tayebi N., Cushner S.R., Kleijer W., Lau E.K., Damschroder-Williams P.J., Stubblefield B.K., Hollander J.D., Sidransky E. (1997). Prenatal lethality of a homozygous null mutation in the human glucocerebrosidase gene. Am. J. Med. Genet..

[41] Carvalho G.B., Ja W.W., Benzer S. (2009). Non-lethal PCR genotyping of single Drosophila. BioTechniques.

[42] Folch J., Lees M., Stanley G.H.S. (1957). A simple method for the isolation and purification of total lipides from animal tissues. J. Biol. Chem..

[43] Inagaki H.K., Kamikouchi A., Ito K. (2009). Methods for quantifying simple gravity sensing in Drosophila melanogaster. Nat. Protoc..

[44] Abraham M.J., Murtola T., Schulz R., Páll S., Smith J.C., Hess B., Lindahl E. (2015). GROMACS: High performance molecular simulations through multi-level parallelism from laptops to supercomputers. SoftwareX.

[45] Meivar-Levy I., Horowitz M., Futerman A.H. (1994). Analysis of glucocerebrosidase activity using *N*-(1-[14C]hexanoyl)-d-*erythro*-glucosylsphingosine demonstrates a correlation between levels of residual enzyme activity and the type of Gaucher disease. Biochem. J..

[46] Kok J.W., Babia T., Klappe K., Hoekstra D. (1995). Fluorescent, short-chain C6-NBD-sphingomyelin, but not C6-NBD-glucosylceramide, is subject to extensive degradation in the plasma membrane: Implications for signal transduction related to cell differentiation. Biochem. J..

[47] Farfel-Becker T., Vitner E.B., Kelly S.L., Bame J.R., Duan J., Shinder V., Merrill A.H., Dobrenis K., Futerman A.H. (2013). Neuronal accumulation of glucosylceramide in a mouse model of neuronopathic Gaucher disease leads to neurodegeneration. Hum. Mol. Genet..

[48] Heyworth R., Dahlqvist A. (1962). Pig intestinal β-glucosidase activities II. Evidence for the hydrolysis of 4-methylumbellifery β-d-glucoside and β-d-galactoside at the same enzyme site. Biochim. Biophys. Acta.

[49] Economos A., Lints F. (1986). Developmental Temperature and Life Span in Drosophila melanogaster. Gerontology.

[50] Chen J., Nolte V., Schlötterer C. (2015). Temperature Stress Mediates Decanalization and Dominance of Gene Expression in Drosophila melanogaster. PLoS Genet..

[51] Maor G., Rencus-Lazar S., Filocamo M., Steller H., Segal D., Horowitz M. (2013). Unfolded protein response in Gaucher disease: From human to Drosophila. Orphanet J. Rare Dis..

[52] Maley F., Trimble R.B., Tarentino A.L., Plummer T.H. (1989). Characterization of glycoproteins and their associated oligosaccharides through the use of endoglycosidases. Anal. Biochem..

[53] Trimble R.B., Tarentino A.L. (1991). Identification of distinct endoglycosidase (endo) activities in Flavobacterium meningosepticum: Endo F1, endo F2, and endo F3. Endo F1 and endo H hydrolyze only high mannose and hybrid glycans. J. Biol. Chem..

[54] Zhao F., Jia C., He F., Hu M., Guo X., Zhang J., Feng X. (2023). Site-Specific Profiling of N-Glycans in Drosophila melanogaster. Front. Biosci..

[55] Braunstein H., Maor G., Chicco G., Filocamo M., Zimran A., Horowitz M. (2018). UPR activation and CHOP mediated induction of *GBA1* transcription in Gaucher disease. Blood Cells Mol. Dis..

[56] Vallerie S.N., Hotamisligil G.S. (2010). The Role of JNK Proteins in Metabolism. Sci. Transl. Med..

[57] Stöven S., Ando I., Kadalayil L., Engström Y., Hultmark D. (2000). Activation of the *Drosophila* NF-κB factor Relish by rapid endoproteolytic cleavage. Embo Rep..

[58] Shaukat Z., Liu D., Gregory S. (2015). Sterile Inflammation in *Drosophila*. Mediat. Inflamm..

[59] Ugur B., Chen K., Bellen H.J. (2016). Drosophila tools and assays for the study of human diseases. Dis. Models Mech..

[60] Babajani G., Tropak M.B., Mahuran D.J., Kermode A.R. (2012). Pharmacological chaperones facilitate the post-ER transport of recombinant N370S mutant β-glucocerebrosidase in plant cells: Evidence that N370S is a folding mutant. Mol. Genet. Metab..

[61] Jung O., Patnaik S., Marugan J., Sidransky E., Westbroek W. (2016). Progress and potential of non-inhibitory small molecule chaperones for the treatment of Gaucher disease and its implications for Parkinson disease. Expert Rev. Proteom..

[62] Beeh K.M., Beier J., Esperester A., Paul L.D. Antiinflammatory properties of ambroxol. 2008, 13, 557–562.

[63] Cabasso O., Paul S., Maor G., Pasmanik-Chor M., Kallemeijn W., Aerts J., Horowitz M. (2021). The Uncovered Function of the *Drosophila GBA1a*-Encoded Protein. Cells.

[64] Pokorna S., Khersonsky O., Lipsh-Sokolik R., Goldenzweig A., Nielsen R., Ashani Y., Peleg Y., Unger T., Albeck S., Dym O. (2023). Design of a stable human acid-β-glucosidase: Towards improved Gaucher disease therapy and mutation classification. FEBS J..

[65] Zhang X.-H., Tee L.Y., Wang X.-G., Huang Q.-S., Yang S.-H. (2015). Off-target effects in CRISPR/Cas9-mediated genome engineering. Mol. Ther. Nucleic Acids.

[66] Xu Y.-H., Quinn B., Witte D., Grabowski G.A. (2003). Viable Mouse Models of Acid β-Glucosidase Deficiency. Am. J. Pathol..

[67] Liu Y., Suzuki K., Reed J.D., Grinberg A., Westphal H., Hoffmann A., Döring T., Sandhoff K., Proia R.L. (1998). Mice with type 2 and 3 Gaucher disease point mutations generated by a single insertion mutagenesis procedure (SIMP). Proc. Natl. Acad. Sci. USA.

[68] Mizukami H., Mi Y., Wada R., Kono M., Yamashita T., Liu Y., Werth N., Sandhoff R., Sandhoff K., Proia R.L. (2002). Systemic inflammation in glucocerebrosidase-deficient mice with minimal glucosylceramide storage. J. Clin. Investig..

