# Supplementary material for: Functional Analysis of Human GBA1 Missense Mutations in Drosophila: Insights into Gaucher Disease Pathogenesis and Phenotypic Consequences
Source: Cells. 2024 Sep 27;13(19):1619. doi: 10.3390/cells13191619 (PMC11475061; doi:10.3390/cells13191619)
Supplement: Supplementary file 1 [file cells-13-01619-s001.zip › cells-3161024-supplementary.pdf]

Figure S1: 4-MUG activity assay and substrate accumulation.

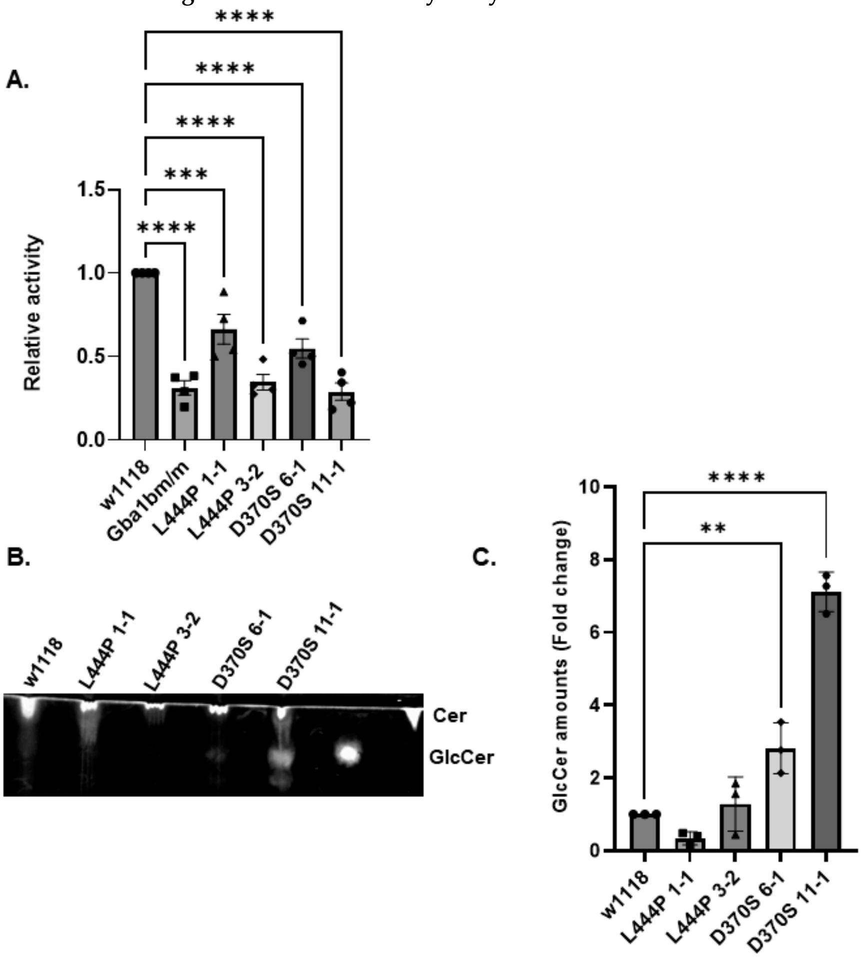

A. 4-MUG activity tested on lysates prepared from w1118, *Gba1b*$^{m/m}$, *Gba1b*$^{L444P/L444P}$ (lines 1-1, 3-2) and *Gba1b*$^{D370S/D370S}$ (lines 6-1, 11-1) flies as detailed under "Methods". Results are presented as average ± standard error. One-Way ANOVA was used to calculate the statistical significance. B. TLC plate showing substrate accumulation in lysates prepared from homozygous flies grown at 29°C for 22 days. C. Quantification of results as shown in (C). The results are presented as average ± standard error. One-Way ANOVA was used to calculate the statistical significance. **p<0.01, ****p<0.001.

Figure S2: Fly pathology upon ambroxol treatment.

A.

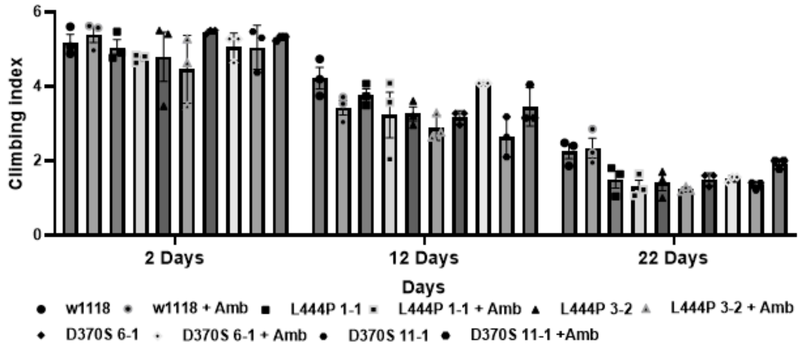

B.

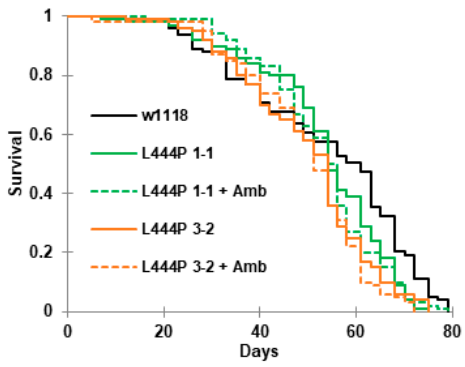

Comparisons	P- value
1-1 vs 1-1 + Amb	1.000
3-2 vs 3-2 + Amb	1.000

C.

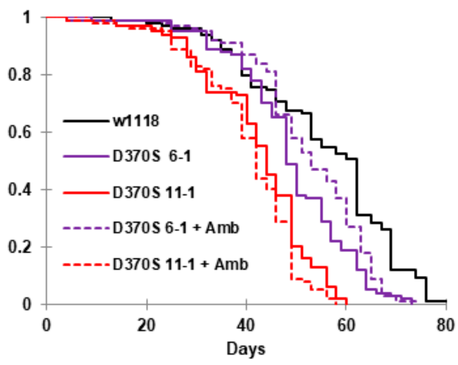

Comparisons	P- value
6-1 vs 6-1 + Amb	0.261
11-1 vs 11-1 + Amb	0.597

A. Thirty flies from *Gba1b*$^{L444P/L444P}$ lines 1-1, 3-2 and *Gba1b*$^{D370S/D370S}$ lines 6-1,11-1, treated or not treated with ambroxol, were tested for their locomotion ability on days 2, 12, and 22 post-eclosion. Results are presented as average ± standard error. Two-way ANOVA was used to determine the statistical significance of the results. B. Kaplan Meier curves presenting the survival of w1118, homozygous *Gba1b*^{L444P} lines 3-2 and 1-1 flies (100 flies from each line), grown with or without ambroxol. Below is a table showing the significance of ambroxol treatment measured by Kaplan Meier's multiple comparisons. C. Kaplan Meier curve presenting the survival of w1118, homozygous *Gba1b*^{D370S} lines 6-1 and 11-1 flies (100 flies from each line) grown with or without ambroxol. Below is a table showing the significance of ambroxol treatment, measured by Kaplan Meier's multiple comparisons.
